# Connection of molecular and cellular components of the immune system in endogenous psychoses with depressive-delusional symptoms

**DOI:** 10.1192/j.eurpsy.2024.585

**Published:** 2024-08-27

**Authors:** S. A. Zozulya, Z. V. Sarmanova, I. N. Otman, I. V. Oleichik, E. G. Cheremnykh, T. A. Prokhorova, O. K. Savushkina, I. S. Boksha, E. B. Tereshkina, T. P. Klyushnik

**Affiliations:** Mental Health Research Centre, Moscow, Russian Federation

## Abstract

**Introduction:**

The data of current research indicate the participation of systemic inflammation in the pathogenesis of endogenous psychoses. Changes in the level of peripheral immune markers are associated with the development of neuroinflammation and correlate with the severity of psychopathological symptoms detected in patients. However, the association between individual components of the immune system involved in the development of endogenous psychosis remains poorly understood.

**Objectives:**

To study the connection between molecular and cellular components of the immune system in women with endogenous psychoses with depressive-delusional symptoms.

**Methods:**

32 female patients aged 23 [17; 36] years with endogenous psychoses within different nosologies (F20, F21, F31, depressive-delusional conditions) and 17 women without clinical signs of psychiatric pathology were examined. The activity of leukocyte elastase (LE), α1-proteinase inhibitor (α1-PI), the proportion of four subpopulations of monocytes (classical CD14++CD16-, intermediate CD14++CD16+, nonclassical CD14+CD16+ and transitional CD14+CD16-) in plasma, activity of cytochrome-c oxidase (COX), glutamate dehydrogenase (GDH), glutathione s-transferase (GST) and glutathione reductase (GR) in platelets and functional activity of complement system (faCS) in serum were determined. The PANSS scale was used to assess the severity of psychopathological symptoms.

**Results:**

Increased activity of the inflammatory markers LE (p=0.033) and α1-PI (p=0.02) was found in the plasma of the patients. Increased percentage of pro-inflammatory monocytes (intermediate and transient) in plasma (p=0.003) was confirmed by negative correlations between CD14++CD16- and CD14++CD16+ (R=-0.685, p=0.00002), CD14++CD16- and CD14+CD16- (R=-0.608, p=0.0002), CD14++CD16- and CD14+CD16+ (R=-0.424, p=0.002). A decrease in GDH activity (p=0.0079), GST activity (p=0.002) and GR activity (p=0.0006) was observed in patient platelets, which can reflect changes in the activity of intracellular metabolic pathways. A positive correlation was found between COX activity and α1-PI (R=0.51, p=0.025). A significant decrease in faCS compared to control (p=0.0003) and a negative correlation between faCS and GST activity (R=-0.496, p=0.011) were observed. faCS was positively correlated with the degree of reduction in the PANSS score (R=0.416, p=0.038).

**Conclusions:**

The revealed connection between molecular and cellular components of the immune system in patients with endogenous psychoses reflect activation of the systemic inflammatory response accompanied by changes in the ratio of monocyte subpopulations and impaired regulation of the complement system. The data obtained can be used to develop methods of monitoring patients taking into account their immunological features.

**Disclosure of Interest:**

None Declared

